# Trends in severe cutaneous adverse drug reactions before, during, and after the COVID-19 pandemic: A retrospective multicenter study of 794 cases

**DOI:** 10.1016/j.jdin.2026.06.006

**Published:** 2026-06-19

**Authors:** Suiting Ao, Qing Zhao, Pengcheng Huai, Lele Sun, Wenchao Li, Yanhong Sun, Xiaoqi Yang, Yonghu Sun, Hong Liu, Furen Zhang, Shandong Severe Cutaneous Adverse Reactions Study Group, Zhongxiang Shi, Baoqi Yang, Qing Yang, Gongqi Yu, Ningning Dang, Weiqing Zheng, Changyuan Wang, Lei Ma, Guowei Zhao, Xiujuan Xia, Liqian Liu, Liang Gao, Zhenhua Wang, Guanzhi Chen, Zhichao Liu, Linlin Xin, Youcan Zhang, Jun Yang, Weiyuan Ma, Haili Ma, Jing Huang, Chenglin Yang

**Affiliations:** a Dermatology Hospital of Shandong First Medical University, Jinan, Shandong Province, PR China; b Shandong Provincial Institute of Dermatology and Venereology, Shandong Academy of Medical Sciences, Jinan, Shandong Province, PR China

**Keywords:** combination therapy, coronavirus disease-19, infection, pandemic, severe cutaneous adverse drug reactions

To the Editor:

Severe cutaneous adverse drug reactions (SCARs) are critical and fatal diseases, such as drug reaction with eosinophilia and systemic symptoms (DRESS) and Stevens-Johnson syndrome/toxic epidermal necrolysis (SJS/TEN), whose mortality rates are up to 40%, as well as other severe forms such as acute generalized eruptive pustulosis and erythrodermic drug eruption.^1^ The relationship between infections and SCARs has garnered widespread attention, including protective and exacerbating effects.^2^^,^^3^ The unprecedented scale of COVID-19 pandemic provides an opportunity to investigate the role of infection in modulating SCARs.

We conducted a multicenter retrospective study across 40 tertiary dermatology departments (January 1, 2019, to December 31, 2023). All SCAR cases were independently confirmed by 2 senior dermatologists. Demographic data and clinical information were stratified into prepandemic (January 1, 2019, to December 31, 2019), intrapandemic (January 1, 2020, to December 31, 2022), and postpandemic (January 1, 2023, to December 31, 2023) periods based on China’s COVID-19 policies and analyzed using χ^2^ and *t* tests.

A total of 794 SCAR cases were recruited, including SJS/TEN (332; 41.81%), erythrodermic drug eruption (304; 38.29%), acute generalized eruptive pustulosis (101; 12.72%), and DRESS (57; 7.18%), with a median age of 52 years and 363 cases occurring in men (Table I). The proportion of DRESS and SJS/TEN cases increased during and after the pandemic (*P* = .0003) (Table I). Autoimmune history was associated with higher odds of SCARs during the pandemic period (adjusted odds ratio [aOR] = 4.55; *P* = .045) (Fig 1). A total of 994 suspected drug reports were identified, as 132 patients had multiple suspected drugs. Antibiotics (334; 33.60%) were the most frequent triggers, followed by traditional Chinese medicines (162; 16.30%), psychotropic drugs (157; 15.79%), antipyretic analgesics (141; 14.19%), and antineoplastic drugs (51; 5.13%). Multiple suspected drugs were involved in a higher proportion of cases after the COVID-19 pandemic (*P* = .0192) (Supplemental Fig, available via Mendeley at https://data.mendeley.com/datasets/jrvtmk793c/1). Multivariable logistic regression showed higher postpandemic attribution for antipyretic analgesics (prepandemic reference: aOR = 1.90, *P* = .046; intrapandemic reference: aOR = 1.54, *P* = .034) and antineoplastics drugs (prepandemic reference: aOR = 4.74, *P* = .037; intrapandemic reference: aOR = 7.07, *P* = .009) (Fig 1).

**Table I:** Demographic and clinical characteristics of SCAR before, during, and after the COVID-19 crisis

	Total	Prepandemic (2019)	Intrapandemic (2020-2022)	Postpandemic (2023)	*P*
Numbers	794	114	359	321	-
Age, y (IQR)	52.00 (36.50-63.00)	50.00 (32.00-81.00)	52.00 (37.00-63.00)	53.00 (37.25-64.00)	.1326
Sex (male:female)	363:431	28:29	158:201	149:172	.1956
Subtype of SCARs					.0003
AGEP	101 (12.73%)	20 (17.54%)	60 (16.71%)	21 (6.54%)	
ED	304 (38.29%)	51 (44.74%)	118 (32.87%)	135 (42.06%)	
DRESS	57 (7.18%)	5 (4.39%)	26 (7.24%)	26 (8.1%)	
SJS/TEN	332 (41.81%)	38 (33.33%)	155 (43.18%)	139 (43.3%)	
Pre-existing diseases					
Hypertension	187 (23.55%)	25 (21.93%)	71 (19.78%)	91 (28.35%)	.0286
Diabetes	79 (9.95%)	15 (13.16%)	35 (9.75%)	29 (9.03%)	.4435
Autoimmune diseases	50 (6.30%)	2 (1.75%)	32 (8.91%)	16 (4.98%)	.0106
Liver diseases	18 (2.27%)	5 (4.39%)	6 (1.67%)	7 (2.18%)	.5054
Malignancy	68 (8.56%)	7 (6.14%)	30 (8.36%)	31 (9.66%)	.2350
Fever (≥38.5 °C)	231 (29.09%)	33 (28.95%)	112 (31.20%)	86 (26.79%)	.4501
Complications					
Hepatic disturbances	161 (20.28%)	18 (15.79%)	72 (20.06%)	71 (22.12%)	.3492
Renal disturbances	13 (1.64%)	2 (1.75%)	7 (1.95%)	4 (1.25%)	.5325
Cardiac disturbances	10 (1.26%)	1 (0.88%)	5 (1.39%)	4 (1.25%)	.9913
Others	21 (2.64%)	3 (2.63%)	10 (2.79%)	8 (2.49%)	.9720
Symptom-to-decision time (d)	5.00 (3.00-10.00)	4.00 (3.00-7.00)	5.00 (3.00-10.00)	5.00 (3.00-9.00)	.0152
Length of hospitalization (d)	9.00 (6.00-13.00)	8.00 (6.00-10.40)	9.00 (7.00-14.00)	9.00 (6.00-13.00)	.3051
Hospitalization expenses (Yuan)	6394.00 (3651.00-10474.00)	5749.00 (2993.00-8546.00)	7389.00 (4346.00-12296.00)	5616.00 (3368.00-8883.00)	<.0001
Death during hospitalization	4 (0.50%)	0 (0.00%)	3 (0.84%)	1 (0.31%)	-

*AGEP*, Acute generalized eruptive pustulosis; *DRESS*, drug reaction with eosinophilia and systemic symptoms; *ED*, erythrodermic drug eruption; *SCARs*, severe cutaneous adverse drug reactions; *SD,* standard deviation; *SJS*, Stevens-Johnson syndrome; *TEN*, toxic epidermal necrolysis.

**Fig 1:**
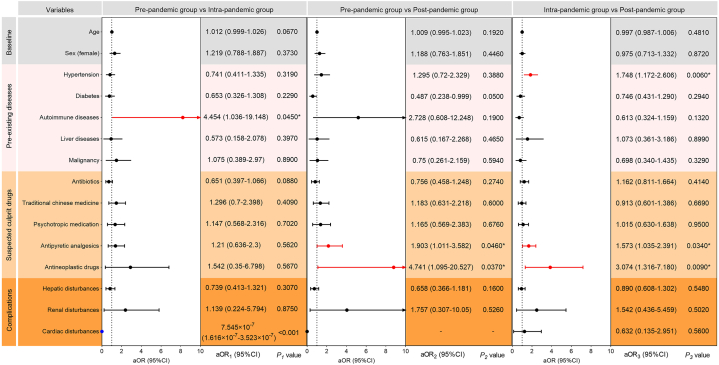
Differences in suspected drug attribution among SCAR cases from multivariable logistic regression (pairwise comparisons across 3 pandemic phases) adjusted for sex, age, pre-existing diseases, culprit drug categories, and complications. *aOR,* adjusted odds ratio; *CI*, confidence interval; *SCARs,* Severe cutaneous adverse drug reactions.

Corticosteroids, immunomodulators, tumor necrosis factor-α blockers, and Janus kinase inhibitors have been used for treating patients with SCAR. Combination therapy was used more often after the pandemic onset (*P* = .0448). Delays in seeking care and higher expenses were found during the pandemic (Table I).

This study demonstrates temporal changes in SCAR phenotypes, culprit drug profiles, and clinical characteristics across the 3 phases of the COVID-19 pandemic. Multiple factors, including health care-seeking changes, care access barriers, and shifts in comedication prescribing, likely contributed. Although the pandemic period is not equivalent to infection, the impact of infection on SCARs cannot be ignored. Long COVID-19 has been associated with altered cytokine profiles from T cells.^4^ Additionally, virus-specific memory T cells can recognize neoantigens generated by drug exposure.^5^ Infection may enhance drug-specific T-cell activation.^5^ Our finding also revealed an increased frequency of severe SCAR phenotypes during and after the pandemic.

We acknowledge the limited sample sizes for DRESS and the prepandemic period, which may affect statistical power. Further research extending beyond 2023 is needed to increase sample size.

In conclusion, temporal shifts in SCAR phenotypes and culprit drugs were observed across pandemic phases, thereby complicating clinical management. Future studies are needed to elucidate pathogenic mechanisms and the impact on SCARs by other viruses.

## Conflicts of interest

None disclosed.
